# Association between Periodontopathogens and CRP Levels in Patients with Periodontitis in Serbia

**DOI:** 10.5681/joddd.2011.003

**Published:** 2011-03-18

**Authors:** Ana Pejcic, Ljiljana Kesic, Jelena Milasin

**Affiliations:** ^1^ Teaching assistent, Department of Periodontology and Oral Medicine, Medical Faculty, University of Nis, Serbia; ^2^ Professor, Department of Periodontology and Oral Medicine, Medical Faculty, University of Nis, Serbia; ^3^ Profesor, Institute of Biology and Human Genteics, Dental Faculty, University of Belgrade, Serbia

**Keywords:** A. actinomycetemcomitans, C-reactive protein, P. gingivalis, periodontitis

## Abstract

**Background and aims:**

Recent epidemiological studies have shown that individuals with periodontitis have a significantly higher risk of developing coronary heart disease, which might be attributed to the complex microbiota in the dental plaque. Periodontopathogens have been reported as risk factors for cardiovascular disease. This study evaluated association of chronic periodontitis and periodontopathogens with CRP in systemically healthy Serbian adults.

**Materials and methods:**

Serum C-reactive protein levels were measured in 24 patients with moderate periodontitis, 26 patients with severe periodontitis, and 25 periodontally healthy subjects. Periodontal health indicators included gingival bleeding on probing and periodontal disease status. Patients with moderate periodontitis had low attachment loss and pocket depths of <4 mm. Patients with severe periodontitis had high AL and pocket depth of >5 mm. The control group with healthy gingiva had gingival sulcus of <2 mm and no attachment loss. Presence of periodontopathogens in subgingival plaque samples was analyzed by polymerase chain reaction.

**Results:**

The periodontal parameters and CRP levels were significantly higher in the patients with periodontitis. Patients who had both severe and moderate periodontitis had higher mean CRP levels. The percentage of subjects with elevated CRP leves of >5 mol/L was greater in the higher clinical AL group compared to the group with less attachment loss. Presence of periodontopathogens was also associated with elevated CRP levels and poor periodontal status.

**Conclusion:**

PD and subgingival periodontopathogens are associated with increased CRP levels. These findings suggest that periodontal infection may contribute to systemic inflammatory burden in otherwise healthy individuals.

## Introduction

Periodontitis (PD) has recently been identified as a potential risk factor for systemic pathologic conditions such as cardiovascular disease. The hypothesis is that local periodontitis, triggered by bacterial insult, is a chronic inflammmatory disease and that production of circulating cytokines may contribute to the development of atherosclerosis and cardiovascular disease among patients with periodontitis.^1,2^ Although periodontitis is chronic in nature, acute-phase elements are also part of the innate immunity in periodontitis and confirm that a systemic inflammation is present in periodontitis.^3^

Unrecognized infections, such as periodontal disease, may induce an acute-phase response, elevating CRP levels.^4,5^ Most studies examined the relationship between periodontitis and CRP in people of different races and nationalities.^6^ Whether this association exists in a Serbian populations is not well documented. We hypothesized that PD may result in an enhanced systemic inflammatory response with higher CRP levels in Serbian population in Nis.

The concept that inflammation plays an important part in the pathophysiology of atherosclerosis and its complication and coronary artery disease has provided a new unifying hypothesis of the links between risk factors and the cellular and molecular alterations that underlie this disease.^7^ This new mechanistic insight has already begun to translate into changes in clinical practice. The preponderance of available data supports the predictive power of biomarkers of inflammation such as C-reactive protein (CRP) in broad categories of individuals, both those as apparently healthy and those with alredy-manifest atherosclerotic cardiovascular disease. The demonstrated clinical utility of CRP and potential of other inflammatory biomarkers has engendered intense interest in evaluating their effectiveness as predictive conventional risk markers and as goals for therapy. The insight that CRP and inflammation play a fundamental role in atherosclerosis and in the pathophysiology of coronary artery disease may lead to novel therapies that target aspects of the inflammatory process smoldering within the atheroma.^8^ CRP reflects activation of the inflammatory process and plays a role in predicting first coronary events in combination with other risk profile factors.^7,9,10^ Moderately elevated serum C-reactive protein (CRP > 2 mol/L) level is a systemic marker of inflammation and a documented risk factor for cardiovascular disease in otherwise healthy individuals. Elevated C-reactive protein values have also been associated with other diseases.^11,12^

Periodontal pathogens, such as* Porphyromonas gingivalis* (*P.g.*) and *Aggregatibacter actinomycetemcomitans* (*A.a.*) are the most important Gram-negative pathogens in the pathogenesis of periodontitis.^13^ Several studies have investigated antibodies to various periodontal pathogens in relation to CRP^14^ but evidence is sparse on the association between a direct measure of periodontal pathogens (*P.g.* and *A.a.*) and CRP.

In this study, a first aim was to evaluate levels of CRP in patients with periodontitis, as well as to examine whether CRP plasma level is in a relation to severity of periodontitis and presence of pathogens in a Serbian population in Nis.

## Materials and Methods

Seventy-five individuals were included in this clinical prospective study: fifty subjects with periodontitis (27 females and 23 males) and 25 subjects without periodontitis (18 women and 7 men).

The patients, who referred to the Department of Periodontology at the Dental Clinic in Nis for the first time to receive treatment for periodontitis, were chosen for this study. After being informed on the purpose of the study, all the patients submitted informed consent forms. The study protocol was approved by the Ethics Committee of Medical Faculty in Nis, Serbia (No:01-2800-5).

Exclusion criteria was any dental treatment during the past 6 months and past smoking habits. Patients were also free from other infections, inflammatory diseases, diabetes mellitus, malignancy, CVD and anti-inflammatory and systemic antibiotic treatments during the previous 3-6 months.

All the clinical examinations were carried out by one examiner. The patients were examined and grouped according to their periodontal status. This classification was based on an original index of severity of periodontal disease based on several references.^2,15 ,16^ Thus, the patients were classified into two groups: Group I (n=24) consisted of patients with moderate periodontitis with pocket depths of <4 mm and moderate mean clinical attachment loss of 2.05 mm. Group II (n=26) consisted of patients with severe periodontal disease with pocket depths of >5 mm and high mean attachment loss of 3.78 mm. Patients who had pocket depths beetwen 4 and 5 mm were excluded from the study.

In both groups, all dental variables were assessed at four different sites (vestibular, mesial, distal and lingual/palatal sites) around each tooth. Dental variables^16^ were:

- Löe-Silness plaque index: PI
- Löe-Silness gingival index: GI
- Bleeding on probing: If bleeding occurred immediately after probing it was reported as positive.The gingival bleeding index was categorized into:

1. no bleeding
2. bleeding

- Periodontal probing depth (PPD) was measured with a standard periodontal probe (Michigen O). Periodontal pocket depth <4 mm (moderate periodontitis) and >5 mm (severe periodontitis) were measured as the distance in millimeters from the free gingival margin to the bottom of the pocket.
- Attachment loss was measured with a standard periodontal probe with 1-mm markings as the distance in millimeters from the cemento-enamel junction to the bottom of the pocket. The values up to 3 mm were labelled as moderate periodontitis and values >3 mm were labelled as severe periodontitis.

In the control group (healthy gums) gingival index was less than 1, gingival sulcus depth was normal (<2 mm). In this group there is no loss of epithelial attachment.

Serum CRP concetration was quantified in milligrams per liter using radial immunodiffusion assay. Samples of peripheral venous blood were taken to determine CRP levels according to the statndard methods in the morning hours, and were processed in the Central Laboratory for Biochemical Researches, Clinical Center Nis. Thus, for study purposes, CRP levels were used to divide patients into low- (CRP concentrations <5 mol/L) and high-level (CRP concentrations >5 mol/L) categories according to te data provided by the Central Clinical Biochemical Laboratory of University in Nis.

The presence of periodontal pathogens * Porphyromonas gingivalis* and *Aggregatibacter actinomycetemcomitans* in subgingival plaque samples was measured by PCR metod. Subgingival plaque samples were obtained from the deepest subgingival sites of periodontal pockets from each patient. The plaque samples were collected with periodontal probe (Michigen O). Plaque samples were suspended in 1 mL of sterile distilled water, palleted, and resuspended in 200 µL of DNA isolation reagent. The suspension was certifuged and 5 µL of the resultant supernatant was used for PCR. The procedures were carried out as described earlier^17,18^ using oligonucieotide primers specific for P.g. and A.a. The PCR product was analysed by 1% agarose gel electrophoresis.

Demographic variables included age, sex, the body mass index (weight-kg/height-m^2^) and smoking status. The body mass and smoking history were self-reported by the patients.

### Statistical analysis

The characteristics of the study groups were analyzed using chi-square test for categorical data, and analysis of variance for continuous data. Differences in CRP levels among groups was evaluated by a post hoc Dunnett test T_3_. Potential confounders were evaluated by using the change-in-estimate method. Potential confounders considered in these analyses were gender, body mass index (BMI) and smoking status.

## Results

Characteristics of the study samples were different in several characteristics, which are presented in Table 1.

**Table 1:** Periodontopathogens and C-reactive protein of the subjects by periodontal status

		Periodontitis (PD) group				
		Moderate (m)	Severe (s)	Control (c)	Total	Pearson Chi-Square
Total						
	Count	24	26	25	75	
	%	32.0%	34.7%	33.3%	100.0%	
P.g.						
Not present	Count	11	6	25	42	
	%	14.7%	8.0%	33.3%	56.0%	χ^2^=32.087
Present	Count	13	20		33	p<0.001
	%	17.3%	26.7%		44.0%	
A.a.						
Not present	Count	18	16	25	59	
	%	24.0%	21.3%	33.3%	78.7%	χ^2^=11.517
Present	Count	6	10		16	P<0.003
	%	8.0%	13.3%		21.3%	
CRP (mol/L)						
≤5	Count	14	12	24	50	
	%	18.7%	16.0%	32.0%	66.7%	χ^2^=15.353
>5	Count	10	14	1	25	p<0.001
	%	13.3%	18.7%	1.3%	33.3%	

P.g.: Porphyromonas gingivalis; A.a.: Aggregatibacter actinomycetemcomitans; CRP: C-reactive protein.

Fifty subjects with periodontitis (27 females and 23 males, mean age of 41.33±17.42) and 25 subjects without periodontitis (18 females and 7 males, mean age of 26.28±3.63) were evaluated.

Patients with periodontitis were older (severe periodontitis: 49.69±16.49 years of age; moderate periodontitis: 47.96±17.21 years of age), had a higher body mass index (severe periodontitis: 24.82±4.01; moderate periodontitis: 23.83±3.39), compared with patients without periodontitis (age: 26.28±3.63; BMI: 21.59±3.90). When analyzed as continuous variables, each indicator of periodontal health might be associated with body mass index.

Table 2. shows that mean CRP levels were significantly higher (8.25 mol/L) among subjects with periodontal pockets (with pocket depths >5 mm), approximately one-half greater than the patients with less periodontal pocket depths (CRP: 4.93 mol/L, pocket depth <4 mm). Mean serum CRP levels were significantly higher in patients with PD when compared to healthy controls (1.09 mol/L) (p<0.001).

**Table 2:** Clinical parameters of the subjects by periodontal status

	Post Hoc Test Dunnett T3	Moderate (m) PD, Mean ± SD	Severe (s) PD, Mean ± SD	Control group (c), Mean ± SD	Total, Mean ± SD	ANOVA
PI	m#c s#c	1.77±0.44	1.96±0.62	1.58±0.56	1.85±0.54	F=58.650 p<0.001
GI	m#c s#c	1.67±0.48	1.82±0.39	1.65±0.49	1.75±0.44	F=103.795 p<0.001
BOP	m#c s#c	1.69±0.47	1.88±0.33	1.70±0.47	1.69±0.47	F=86.971 p<0.001
PPD	c # m #s	3.82±0.57	5.27±0.70	1.76± 0.39	3.64±1.57	F=244.286 p<0.001
AL	c # m #s	2.05±0.53	3.78±0.83	0.25±0.19	2.75±1.29	F=68.471 p<0.001
CRP (mol/L)	m ~ s c#m c#s	4.93±3.23	8.25±9.35	1.09±1.48	6.59±7.31	F=10.168 p<0.001
BMI	c ~ m ~ s c#s	23.83±3.39	24.82±4.01	21.59±3.90	23.43±3.98	F=4.858 p=0.010

PD: periodontitis; SD: standard deviation; PI: plaque index; GI: gingival index; BOP: bleeding of probing; PPD: periodontal pocket depth; AL: attachment loss; CRP: C reactive protein; BMI: body mass index.

Subjects with severe periodontitis and high levels of mean clinical attachment loss (3.78 mm) had significantly higher mean CRP levels (8.25±9.35 mol/L) than subjects with moderate periodontitis and lower levels of mean clinical attachment loss (2.05±0.53 mm) and mean CRP levels (4.93±3.23 mol/L). In the control group, CRP levels were very low (1.09±1.48 mol/L).

The periodontal parameters (PI, GI, BOP and PPD) were significantly correlated with CRP levels in periodontal patients, especially in the group of patients with severe periodontitis (p<0.001, p<0.001, p<0.001, p<0.0001). Almost 70% of the subjectss had C-reactive protein concentrations lower than 5 mol/L.

The CRP levels were adjusted for factors known to be associated with elevated CRP levels, including gender, smoking, and body mass index (BMI). Gender and BMI were found to be significant covariates (p<0.144, p<0.003).

The presence of periodontal pathogens P.g. and A.a. in subgingival samples was positively associated with elevated CRP levels (P.g.: 44%, A.a.: 21.3 %) (p < 0.001, p<0.001).

## Discussion

Periodontitis has recently been identified as a potential risk factor for systemic pathologies such as cardiovascular disease.^19,20,21^ Poor oral health and coronary heart disease are major worldwide health problems, and their associations are potentially important. In addition to classical risk factors, chronic infection such as periodontal diseases are mentioned as potentional risk factors for coronary heart disease^22,23^

After the initial publication of the results of a study by Mattila et al.^24^ indicating that patients with periodontal infections have significant elevations of plasma fibrinogen and white blood cell counts, a subsequent study observed that periodontitis was also associated with other markers of activated inflammation and hemostasis, including CRP.^25^ The mechanisms by which periodontitis contributes to CVD have not been clearly understood, but there are numerous working hypotheses that will be proved or disproved.^26,27^

The results of this study confirmed those of previous studies conducted on subjects that showed a significant association between dental disease and CVD.^28,2p,30^ These analyses of the data from a sample representative for the Serbian population support the existence of a signaficant relation between periodontal health status of both CRP and periodontal pathogens. Therefore, CRP levels were significantly elevated among individuals with severe periodontitis (8,25 mol/L), and among individuals with moderate periodontitis (4,93 mol/L) than in the controls (1.09 mol/L) (Table 2). CRP levels were approximately 50% higher in people with extensive pockets than those with minimal disease state, and seven times higher than those without disease, after adjusting for tradicional risk factors. In this study statistical analyses were performed with adjustment for potential confounders such as gender, smoking status, and body mass index. These results were consistent with the results of other studies.^6,31,32,33^ The association of periodontits and C reactive protein levels appears to be independent of other contributing factors in patients with PD and increased CRP levels (Table 1), associated significantly with risk of any stroke and risk of ishemic stroke.^34^

A similar significant relation was observed between the presence of P gingivalis and increased CRP levels after adjusting for gender, BMI, and smoking.^35,36,37^ Our study results showed elevated presence of periodontal pathogens such as P.g. in individuals with periodontitis (Table 1).

As shown in Tables 1 and 2, the percentage of subjects with P gingivalis increased from 17.3% in subjects with moderate periodontitis and attachment loss of 2.05±0.53 to 26.7% in subjects with severe periodontitis and attachment loss of 3.78±0.83. Previous reports using these data have suggested that P gingivalis is associated with increasing periodontal disease severity.^38,39^ Also, the association between presence of periodontal pathogens and CRP levels has been established. A link between periodontal pathogens and CRP hasa been observed in relation to periodontal disease. Our results indicate that an elevated P gingivalis count is independently associated with high serum CRP levels. In contrast, A actinomycetemcomitans counts were not associated with CRP levels. The data confirm that both a high extent of attachment loss, representing a widespread periodontitis, and P gingivalis counts are independently associated with elevated CRP levels. An effect of periodontitis, assessed by clinical measures, on high CRP level has been documented in a number of studies (Table 3).^40,31,15^

**Table 3:** Characeristics of the subjects by C-reactive protein (CRP) levels

		CRP≤5(mol/L)	CRP>5(mol/L)	Total	Pearson Chi-Square	Fisher's Exact Test
Total						
	Count	50	25	75		
	%	66.7%	33.3%	100.0%		
Gender						
male	Count	17	13	30		
	%	22.7%	17.3%	40.0%	χ^2^=2.250	p<0.144
female	Count	33	12	45	p<0.134	
	%	44.0%	16.0%	60.0%		
Smoking						
Non-smoker	Count	28	14	42		
	%	37.3%	18.7%	56.0%	χ^2^=.000	p<1.000
Smoker	Count	22	11	33	p<1.000	
BOP						
1	Count	23	3	26		
	%	30.9%	3.3%	34.2%	χ^2^=13.483	
2	Count	27	22	49	p<0.001	
	%	35.8%	30.0%	65.8%		
P.g.						
Present	Count	41	1	42		
	%	54.7%	1.3%	56.0%	χ^2^=41.153	p<0.001
Not present	Count	9	24	33	p<0.001	
	%	12.0%	32.0%	44.0%		
A.a.						
Present	Count	48	11	59		
	%	64.0%	14.7%	78.7%	χ^2^=26.854	p<0.001
Not present	Count	2	14	16	p<0.001	
	%	2.7%	18.7%	21.3%		
		Mean ± SD	Mean ± SD	Mean ± SD	t-test	
BMI		22.48±3.93	25.33±3.41	23.43±3.98	t= -3.088	p<0.003
PPD		3.14±1.54	4.63±1.081	3.67±1.57	t= -4.873	p<0.001
AL		2.494±1.341	3.050±1.193	2,751±1,294	t= -1.600	p<0.116

CRP: C reactive protein; BOP: bleeding on probing; P.g.: Porphyromonas gingivalis; A.a.: Aggregatibacter actinomycetemcomitans; BMI: body mass index; PPD: periodontal pocket depth; AL: attachment loss.

This study was able to indicate that elevated CRP levels tend to be more closely related to gingival bleeding, poor oral hygiene, plaque index, gingival index, and presence of periodontal microorganisms (*P.g., A.a.*) (Table 2). The presence of these microorganisms in this study may at least partially explain why consistent association was observed between periodontal status and CRP levels in comparison with the association with CVD. The proposed aetiological mechanism behind this association was attributed to the effect of oral bacteria on cells taking part in the pathogenesis of atherosclerosis and thrombosis. Thus, even though an association between periodontitis and elevated risk factors (CRP) for CVD were observed with the presence of periodontal microorganisms,^2^ further studies are needed to confirm the possible role of specific harmful microorgamisms in the association observed.

Support for our findings also comes from studies that indicate that several periodontal organisms, including P.g. and A.a. have been detected directly within the atherosclerotic plaque lesion of the vessel wall.^41,42,43^ The loss of epithelial integrity within the periodontal pocket creates direct bacterial translocation and bacteriemia. Sistemic exposure to oral bacteria may lead to elevated circulating cytokines. Previous studies suggest that lipopolysaccharides act as a systemic trigger that can activate an impressive cascade of inflammatory cytokines, eliciting most of the vascular and coagulation complications associated with atheroaclerosis. LPS may lead to the secretion of serum cytokines and inflammatory mediators, which could contribute to the chronic inflammatory process that leads to elevated levels of CRP in serum. LPS has long been known to promote atherosclerosis and thrombus formation.^1,17,29^

CRP, as a systemic marker of inflammation, is a predictive marker for cardiovascular diseases. Therefore, changes in this marker in periodontitis may be part of the explanation why periodontitis is associated with cardiovascular diseases. It is hypothesized that possibly daily episodes of bacteriemia originating from periodontal lesions are the cause for the changes in systemic markers in periodontitis. Although observational studies suggest an association between periodontitis and CRP as a risk factor in cardiovascular disease, the reason for this relationship is not fully understood.

## Conclusion

Researchers have found that people with periodontal disease have increased CRP levels depending on the severity of the disease, and subgingival organisms, including P.g. and A.a. The positive correlation between CRP and periodontal disease might be a possible underlying cause in the association between periodontal disease and the observed higher risk for CVD in these patients.
